# Medicinally Important Herbal Flowers in Sri Lanka

**DOI:** 10.1155/2019/2321961

**Published:** 2019-05-27

**Authors:** S. L. A. Gunawardana, W. J. A. B. N. Jayasuriya

**Affiliations:** Department of Pharmacy and Pharmaceutical Sciences, Faculty of Allied Health Sciences, University of Sri Jayewardenepura, Sri Lanka

## Abstract

**Background:**

The plant kingdom is rich with a numerous number of plants with various medical properties which can be used to treat various medical issues. Sri Lanka is a country full of biodiversity which is gifted with many plant resources. It has a rich history of traditional medicine systems consisting of Ayurveda, Unani, and Deshiya Chikitsa, where these plant resources are used as remedies for the diseases. In the traditional medicine system, various plant parts such as leaves, roots, fruits, flowers, and bark are used to treat disease conditions. Although less attention is paid to the medicinal importance of the flowers, some of them have been used to treat many diseases from the ancient time. Some properties of the flowers may differ from the properties of the other plant parts. For example,* Sesbania grandiflora* (Katuru murunga) flowers have shown anticancer properties against various cell models whereas some flowers have shown antispermatogenic properties. Flowers of* Woodfordia floribunda *(Militta) are added as fermenting agents in the preparation of Arishtas in Ayurveda. Also the most popular Clove oil is obtained from the flower buds of* Syzygium aromaticum *(Karabu-neti) which is used to treat toothaches since it has antibiotic and antiseptic properties. This article gives an overview of herbal flowers used in the traditional medicine system of Sri Lanka and their pharmacological importance.

**Method:**

A comprehensive literature survey was done on the medicinally important flowers in Sri Lanka. Data was collected from Libraries of Ayurveda in Sri Lanka and from scientific databases.

**Results:**

According to the survey many flowers are used as astringent, cardiac tonic, and febrifuge. Also some flowers are used to treat dysentery, diarrhoea, and indigestion. Some flowers are useful in the treatment of bleeding piles while some are useful in the treatment of asthma and bronchitis.

**Conclusion:**

It was revealed that there are many flowers with valuable therapeutic effects. Traditional medicine systems prevailing in Sri Lanka have made use of these flowers with therapeutic effects to cure so many diseases. The review of medicinally important herbal flowers provides knowledge and pharmacological leads which will help for the wellbeing of the human beings. Although there are phytochemical studies done to identify the chemical compounds on some flowers, chemical composition of many flowers remains unrevealed. So further studies need to be done to identify the chemical composition of these flowers.

## 1. Introduction

Plants have been used by human beings to cure diseases from ancient time. Therefore herbal drugs play a major role in traditional medicine. According to the World Health Organization (WHO), herbal drugs are the best source to obtain a variety of drugs. In developing countries, about 80% of the population depends on traditional medicine [1]. The traditional medicine which is mainly based on plant sources consists of significant amounts of bioactive compounds. These compounds provide valuable health effects. Since modern medicine claimed to possess irreversible adverse events, herbal plants play a major role in delivering therapeutic effects with less adverse events to human beings [2].

Sri Lanka is a country full of biodiversity which is gifted with many plant resources. It has been reported that there are 3771 flowering plant species grown in Sri Lanka. Out of them about 927 (24%) are endemic to the country. Also, 1430 species are considered to have medicinal value. Out of these medicinal plants, 174 (12%) are endemic to Sri Lanka. Also, it is reported that around 250 species are commonly used in traditional medicine. Since there are a huge number of medicinal plants in Sri Lanka, it has a rich history of traditional medicine systems [72]. The main traditional medicine systems that prevail in Sri Lanka are Ayurveda, Siddha, Unani, and Deshiya Chikitsa. In Ayurveda, herbal preparations are used mostly to cure diseases while in Siddha mineral preparations are used mostly. Deshiya Chikitsa also uses herbal preparations. The Unani differs from these concepts. Ayurveda system of Sri Lanka nearly uses 2000 herbs [73]. These systems fulfill 60-70% of the rural populations' primary health care needs. The knowledge regarding the use of these herbal plants has been passed from generation to generation in the traditional medicine systems. So there are numerous plants with valuable health benefits used in these traditional formulations where the knowledge regarding the therapeutic use is depleting day by day [72].

In the recent past, there has been a tremendous increase in the research done on herbal medicine and there has been an increase in the use of herbal products in the developing countries and in developed countries. Today nearly 51% of the approved drugs are directly or indirectly derived from the herbal plants [74]. Plants provide the constituents for the synthesis of new drugs and chemical compounds. The secondary metabolites, such as tannins, terpenoids, alkaloids, flavonoids, phenols, steroids, glycosides, volatile oils, etc., also are a major source of therapeutically valuable compounds [75]. These compounds of pharmacological importance can be obtained from various plant parts such as leaves, roots, flowers, bark, etc. [76]. Flowers are reproductive parts of a plant which are also used for nutritive and medicinal properties. Flowers are used either directly or as a decoction, tincture, or mixed with other ingredients to treat diseases [77]. Flowers such as* Stereospermum suaveolens* (Roxb.)/*Bignonia suaveolens* Roxb (Palol) are used to treat malaria and bronchitis [61]. Some flowers such as* Woodfordia floribunda *(Militta) are added as fermenting agents in the preparation of Arishtas in Ayurveda [3]. Also some flowers possess properties that are different from the pharmacological properties of the other parts of the plant. For example, flowers of* Butea monosperma* have diuretic, astringent, and tonic properties while the root of this plant has analgesic properties [18].

According to the literature survey there were numerous review articles about medicinal plants. But only few research papers were focused on the particular parts of the medicinal plants. Therefore the study of pharmacological importance of the flowers is benefitted in the development of novel drugs.

## 2. Methodology

A comprehensive literature survey was done on the medicinally important herbal flowers in Sri Lanka. The data was obtained from the Libraries of Navinna Ayurveda Hospital, Maharagama, Sri Lanka, and Institute of Indigenous Medicine, University of Colombo, Rajagiriya, Sri Lanka. Books like Medicinal Herbs and Flowers [78], The Chemistry and Pharmacology of Ceylon and Indian Medicinal Plants [3], Medicinal Plants Used in Ceylon-Part I [7], Part II [25], Part III [24], Part IV [27], and Part V [51], Sri Lankawe Osu Shaka Saha Ewaye Wadagathkama [8], and Medicinal Herbal [5] were used. Further research was carried out based on the scientific names collected from the flowering ayurvedic plants from these books using scientific databases PubMed, Google Scholar, and Web of Science. Nine Ayurveda books and 69 journal articles were referred in this article.

## 3. Results and Discussion

**Figure 1:**
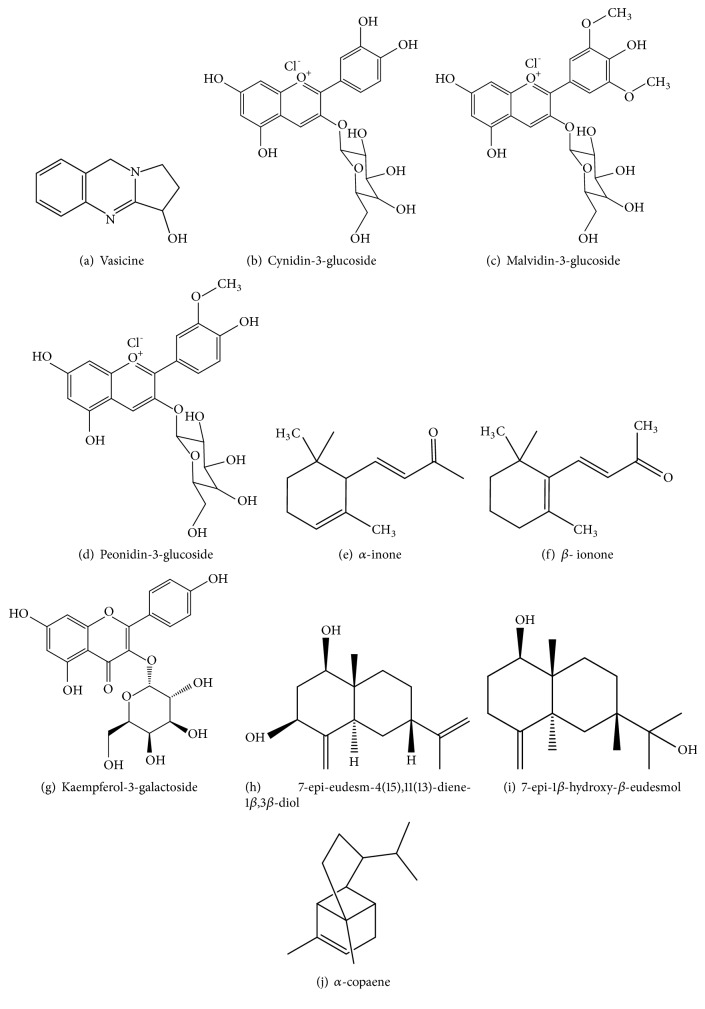
Chemical compounds present in medicinally important flowers [9, 16, 21, 39, 40].

Table 1 includes the information collected from the extensive literature survey and Figure 1 includes some of the chemical structures of the phytochemicals found in Sri Lankan herbal flowers.

In this review an attempt has been taken to present the medicinally important flowers in Sri Lanka and their therapeutic uses. Generally, flowers have similar pharmacological properties as their other parts. But several flowers were reported to have special medicinal uses. Flowers such as* Stereospermum suaveolens* (Roxb.)/*Bignonia suaveolens* Roxb (Palol) flowers are used to treat malaria and bronchitis. Also the most popular Clove oil is obtained from the flower buds of* Syzygium aromaticum *(Karabu-neti) which is used to treat toothaches since it has antibiotic and antiseptic properties. Some flowers such as* Woodfordia floribunda *(Militta) are added as fermenting agents in the preparation of Arishtas in Ayurveda. Also powder of this flower is administered with honey in the treatment of leucorrhoea and water decoction of the fresh flowers of this plant either alone or in combination with ginger (*Zingiber officinale*) is used for the treatment of dysentery. Flowers of* Wrightia antidysenterica* Linn (Sudu idda/Wal-idda) are important for the treatment of Russell's viper snake bite and to treat Gonorrhea. The leaf, flower, and seeds of* Sphaeranthus hirtus/indicus* (Mudamahana) plant are ground into a paste and applied topically to treat skin diseases. Juice of* Saraca indica* (Asoka) flowers are used as a cardiac tonic. Also some flowers possess properties that are different from the pharmacological properties of the other parts of the plant. For example* Butea monosperma* flowers have diuretic, astringent, and tonic properties while the root of this plant has analgesic properties. The flowers of* Punica granatum* had been used in traditional medicine to treat vaginal discharge and diarrhoea while the juice of fruit is used to treat gallbladder diseases.

## 4. Conclusion

An extensive literature survey done on the flowers with medicinal importance in Sri Lanka revealed that there are many flowers with valuable therapeutic effects. Some plants are endemic to Sri Lanka while some are distributed throughout the world. Traditional medicine systems prevailing in Sri Lanka have made use of these flowers with therapeutic effects to cure so many diseases. Considering these facts need was felt to collect details regarding the chemical composition and their ailments. Although there are number of phytochemical studies were carried out using these flowers, exact chemical composition is remain unrevealed. Hence, further studies are warrants on these flowers in order to identify their biological activities, mechanisms of action, and the chemical composition. Further, there is a possibility of developing novel formulations for various diseases for the betterment of the mankind. Thus the review of the medicinally important flowers in Sri Lanka provides details regarding the pharmacological leads for the wellbeing of the human society.

## Figures and Tables

**Table 1:** Medicinally important herbal flowers in Sri Lanka.

Name of the flower	Commonly grown area in Sri Lanka	Traditional Uses	Pharmacological Uses	Chemical composition
*Abutilon indicum/Abutilon asiaticum* English: Country mallow Sinhala: Anoda Tamil: Perunutti	Dry country of Sri Lanka	Local application for the boils and ulcers [3]. Flowers are used to increase the volume of semen in human beings [4].	It has emollient properties [4].	

*Acacia Arabica* Sinhala: Babbula	Along the coast of Sri Lanka	The flowers and pods are used as astringent in diarrhoea, dysentery, spermatorrhoea, leucorrhoea and premature ejaculation in the form of powder or decoction. This is used externally for washing bleeding ulcers and wounds and in reducing body temperature, earache and as a tonic [5].	It has astringent properties [5].	Stearic acid, kaempferol-3-glucoside, isoquercetin and leucocyanidin [6].

*Adhatoda vasica* Sinhala: Pawatta/Adhatoda Tamil: Adadodi/Kattumurungai	Dry country of Sri Lanka	The flowers are regarded as antiseptic, insecticidal and expectorant, used to treat cough, chronic bronchitis and asthma. The fresh flowers are used for ophthalmia [7, 8]. Flowers are given as an infusion to treat fever and used in the treatment of gonorrhea, jaundice, rheumatism and abdominal tumour [9]. These are also used to improve blood circulation and hectic heat of blood [10].	Flowers have bronchodilator, sedative, expectorant, antiseptic and antispasmodic properties [11]. Ethyl acetate extract of these flowers possesses significant antibacterial and antifungal activity against microorganisms like (Bacteria) *Salmonella typhi, Escherichia coli, Escherichia faecalis, B. cereus* and (fungi) *Candida lunata, Candida albicans *[12].	Vasicine (Figure 1(a)), a bitter quinazoline alkaloid and chemical compounds such as triterpenes (a-amyrin), flavonoids (astragalin, kaempferol, quercetin), alkanes [9] and 2′-4-dihydroxychalcone-4-glucoside [13].

*Asclepias curassavica* English: West Indian Ipecacuanha Sinhala: Kankumbala	Commonly found in open waste places, particularly along the sea coasts of Sri Lanka.	The powdered flowers are used for treating sores and wounds [7].		

*Bauhinia variegate* Sinhala: Koboleela		Dried buds of this plant are used for dysentery, piles, worm, diarrhea and tumors. The juice of flower is used to treat diarrhea, dysentery and other stomach disorders [14].	Flowers and flower buds have laxative properties and free radical scavenging activity [15]. The flowers have chemopreventive role against 7,12-dimethylbenz(a)anthracene (DMBA)-induced skin carcinogenesis in mice [14].	The pale while flowers contain cynidin-3-glucoside (Figure 1(b)), malvidin-3-glucoside (Figure 1(c)), malvidin-3-diglucoside and peonidin-3-glucoside (Figure 1(d)) and peonidin-3-diglucoside. The white flowers contain kaempferol-3-galactoside (Figure 1(g)) and kaempferol-3-rhamnoglucoside [16].

*Butea frondosa/monosperma* Sinhala: Kela		The flowers are useful in the treatment of liver disorders, diarrhea, leprosy, leucorrhoea, gout and as a tonic [17].	The flowers possess antistress, diuretic, astringent, depurative, nootropic activities and antifungal activity against *Helminthosporium sativum* [17].	Triterpenes, several flavonoids butein, butin, isobutrin, coreopsin, isocoreopsin (butin 7-glucoside), sulphurein, monospermoside (butein 3-e-D-glucoside) and isomonospermoside, chalcones, aurones, isobutyine, palasitrin, 3′,4′,7-trihydroxy flavone. Myricyl alcohol, stearic acid, palmitic acid, arachidic acid, lignoceric acid, glucose, fructose, histidine, aspartic acid, alanine and phenylalanine [18].

*Boerhaavia diffusa* Sinhala: Pita sudu sarana		The flowers and seeds are used as contraceptives in ayurvedic medicine [19].	Flowers have diuretic and anti-inflammatory activity [19].	

*Ceiba pentandra* English: Silk cotton Sinhala: Imbul/Pulung Tamil: Ilavam, Karukkanam	Very common plant in the low country. They are nearly always planted as fence pots along boundaries in Sri Lanka	The ethanolic extract of the bark and flower is given for three days to treat sexual diseases like leucorrhoea, gonorrhoea and to regulate menstrual abnormalities in women. These are used as an astringent and are used for the treatment of skin troubles, splenomegaly and haemorrhoids. They are given with seeds of *Papaver somniferum*, sugar, and milk to cure piles.	The methanolic extract of the flower possesses hepatoprotective activity. These also possess antioxidant and antiproliferative activity against human several cancer cell lines.	*β*-d-glucoside of *β*-sitosterol, free *β*-sitosterol, hentriacontane, hentriacontanol, traces of essential oil, kaempferol, and quercetin [20].

*Chrysanthemum indicum* Sinhala: Kapuru		These flowers are used to treat stomatitis, burning sensations, urinary discharges, gleet, lumbago, in obstructive affections of the brain and calculus and to remove depression states. It has been used in conjunction with black pepper for gonorrhea. Flower heads infusion is said to be frequently applied as collyrium in eye affections. Also administered for sore eyes and for the internal or external abdominal inflammations. The flowers are applied on the skin to treat leprosy [5]. It also used as a heat-clearing and detoxifying herb [21].	The flowers have antibacterial, antioxidant and anti-inflammatory properties [21]	The main volatile oils present in the flowers are 2,6,6-trimethyl bicycle [3.1.1]hept-2-en-4-ol and 2-(2,4-hexadiynylidene)-1,6-dioxaspiro[4.4]non-3-ene, germacrene D, á-neoclovene, eucalyptol, á-pinene, 1,4-bis(1-methylethyl)-benzene, âsesquiphellandrene, longipinane and 7, 11-dimethyl-3- methylene-1,6,10-dodecatriene. Flavonoids are also present, i.e., quercitrin, myricetin, luteolin-7-glucoside. Vitexin and apigenin are the least abundant flavonoids in the plant [22]. New sesquiterpenoids identified are 7-*epi*-eudesm-4(15),11(13)-diene-1*β*,3*β*-diol (Figure 1(h)) and 7-*epi*- 1*β*-hydroxy-*β*-eudesmol (Figure 1(i)) in the ethanolic extract of the flower [21].

*Cochlospermum gossypium* English: Golden silk cotton Sinhala: Kinihiriya		The powdered flower is given to children in empty stomach to improve immunity and memory power in children.	The flowers consist of stimulant properties [23].	

*Cymbopogon jwarancusa* English: Lemon grass Sinhala: Sera		The paste of root and flower buds is effective in dissolving the hard inflammations in the internal organs. Oil of the flower bud is a good embrocation, also useful in mouth gargles to strengthen the gums. Also effective as a digestive tonic and to stop diarrhoea [24].		

*Eclipta prostrata* English: False daisy Sinhala: Kikirindi Tamil: Kaikeshi		A decoction of the flower tops and leaves is given for hepatitis [25]. Flowers are used for the treatment of urinary problems, jaundice, asthma and coughs [26].	The aqueous extract of the flower has shown antimicrobial activity against *P. vulgaris, S. aureus,* and *S. saprophyticus *[26].	

*Hibiscus rose-sinensis* English: Shoe flower Sinhala: Wadamal/Sapaththumal Tamil: Arattam	These are commonly cultivated in the gardens. It grows well anywhere from the sea coast to the hill country and in the dry zone in Sri Lanka.	Shoe flowers are used as an expectorant for bronchitis, paralysis, dysmenorrhea and cough. Flowers are eaten with papaya seeds to cause abortion. The shoe flower leaves and young flower buds are used as a poultice on boils and swellings.	The flowers possess anti-spermatogenic, androgenic, anti-tumour, anti-diabetic and anticonvulsant activities.	Small amount of hibiscetin [27].

*Hibiscus tiliaceus* Sinhala: Beli-patta Tamil: Nir-paratthi	Very common in the low country of Sri Lanka.	Flowers are boiled in milk to treat earache [27]. The slimy sap of the bark, branches and flower buds are used as a mild laxative or as a lubricant in childbirth or labor pains and rubbed on stomach and to treat bronchitis. The aqueous extract of wood and fresh flowers of this plant is used as a treatment for skin diseases [28].	The flowers consist of emollient properties and anti-depressant like activity [27].	The methanol extract consists of stigmasterol, stigmastadienol and stigmastadienone [29].

*Horsfieldia iryaghedhi* Sinhala: Ruk	Often found in the low-country up to 1000 feet altitude.	The flowers are used to treat dysentery, hiccough and wasting diseases [27].		

*Impatiens balsamina* English: Jewel weed Sinhala: Kuudalu	Grown as a garden plant commonly in Sri Lanka.	Flowers are applied for burns, scalds, lumbago and intercostal neuralgia [30].	The flowers are having cooling, demulcent and tonic effect, anti-anaphylactic and anti-hypotensive properties. The alcoholic extract of flowers has antibiotic activity against, *Fructicola* and other pathogenic fungi and bacteria and the methanolic extract of the flowers has anti-tumour promoting activity, peripheral and central anti-nociceptive activity [31, 32]. It was found that the effects were rapid and long lasting and these effects were mediated by the inhibition of opioid receptors and peripheral inflammatory mediators like cyclooxygenase 2 [33]. The kampherol and quercetin possesses antibacterial activity against *Propionibacterium acnes *[34]. Also, the isolated kampherol has shown mushroom tyrosinase inhibitory activity which proves the fairness activity of the flower extract [32].	Flavanol, kampherol, quercetin, myricetin [35], phenolic compounds, impatienolate, balsaminolate, lawsone methyl ether, kaempferol 3-rutinoside, 2-hydroxy1,4-naphthoquinone, p-coumaric and ferulic acids [33].

*Ipomoea aquatic* English: Water spinach Sinhala: Kangkong Tamil: Kalaka	Very common in shallow water and moist places in the dry region of Sri Lanka.	The bud is applied to treat ringworm infections [25].		

*Ixora coccinea* Sinhala: Ratmal Tamil: Vedchi		The flowers are fried in melted butter, rubbed down with a little cumin and nagakesara and made into a bolus with sugar candy. This preparation is used as a remedy for dysentery [3]. Also, the flowers are famous for its use to treat catarrhal bronchitis, dysmenorrhea, hemoptysis and leucorrhoea in Ayurvedic medicine [32].	The aqueous flower extracts have also shown anti-diarrhoeal activity against a castor oil induced diarrhoea model in rats and alcoholic extract has shown wound healing activity in dead space wound model in rats [36]. The methanolic extract was reported to possess the analgesic, anti-inflammatory, antiulcer, fairness [37] and broad-spectrum anti-microbial activity [38]. Antitumour activity of *I. coccinea* flowers was studied on Dalton's lymphoma (ascitic and solid tumours) and Ehrlich Ascites Carcinoma (EAC) tumours in mice and it was reported that there is a significant antitumour activity in the flower extracts. Also chemoprotective effect of flowers extracts has shown a significant effect on cisplatin induced toxicity in mice [37].	Essential oil extracted from the flowers contains triterpenes, monoterpenes, sesquiterpenes and an ester. The constituents of triterpenes detected are ursolic acid, oleanolic and lupeol while geranyl acetate is detected as the major monoterpene. Further analysis of the methanolic flower extract has revealed the presence of biochin A, myricetin, quercetin, rutin, diadzein and formononetin compounds [37].

*Ixora parviflora* English: Toreb tree Sinhala: Maha ratambala Tamil: Karankutti		The flowers pounded in milk are given in whooping cough [3].		

*Lawsonia alba* English: Henna Sinhala: Marithondii Tamil: Maritondi		An infusion of flowers of this plant is said to be a good application to bruises [3]. Decoction of the flowers is used as an emmenagogue [39].		Essential oil (0.02 %) rich in ionones (90 %) *α*-ionones (Figure 1(e)) and *β*-ionones (Figure 1(f)) in which *β*-ionones predominated [39].

*Mesua ferrea* English: Iron wood tree Sinhala: Na Tamil: Nagacuram		The paste of flowers is applied to bleeding piles and for burning of the feet as a paste made with butter and sugar. Also, they are used against snake and scorpion sting [40]. A syrup of the flower buds is given in treatment of dysentery [3]. A decoction of 2-3 flowers with sugar candy is given twice a day for to stop bloody stool. The flower buds are used to treat sore throat, cough and asthma [40].	Methanolic extract of the flower consists of antioxidant and hepatoprotective activity and methanolic and dichloromethane extract of the flower has shown antimicrobial activity against *Micrococcus luteus*, *E. coil*, *Candida albicans* and *Aspergillus niger*. The flowers have stomachic, expectorant and astringent properties [40].	*α*-copaene (Figure 1(j)) and germacrene D compounds [40].

*Magnolia fuscata* Sinhala: Madana-kama Tam: Madanakam-poo	This plant is cultivated both in the hill country and mid country of Sri Lanka.	The seeds and flowers of this plant are used for making preparations for strengthening sexual virility [27].		

*Melia azedarach* English: Bead tree/Indian Lilac Sinhala: Mahanimba Tamil: Malaivenbu		A paste of flowers is applied on the head to destroy lice and for eruptions of the scalp. The flowers and leaves are applied as a poultice to relieve nervous headaches. The oil of the flower contains sulphur and inorganic combination and is useful for all cutaneous ailments and rheumatism. The flowers are also used to treat cephalalgia, gastropathy, verminosis, strangury, dysmenorrhoea and fever.	According to the studies, the methanolic extract of *M. azedarach* flowers also shows potent antibacterial activity. The flowers have astringent, refrigerant, anodyne, stomachic, vermifuge, diuretic, abortifacient deobstruent and alexipharmic activities [41].	

*Nelumbo nucifera* English: Chinese water lily/Sacred lotus Sinhala: Nelum Tamil: Ambal	Common in the tanks of dry zone of Sri Lanka.	The stamens of the flowers are used bleeding piles and debility and weakness in children. The flowers with stamens and Juice of flower stalk are used for diarrhoea, cholera, fevers, liver complaints, and as a cardiac tonic. The flower is made into syrup and given for coughs, dysentery and to check haemorrhages from bleeding piles. The ground petals are administered for syphilis in Malaya. The milky juice of the leaves and flower stalk is given for diarrhoea. In China and Malaya the dried petals and stamen of the pink flower variety are used as a cosmetic to improve the complexion. Also, the flowers are used as cooling and astringent agents. The flower beverages are used to treat cancer, weakness and body heat balance. Stamens are used to treat consolidation of kidney function, aphrodisiac, male sexual disorders and female leucorrhea. Pounded petals of the flowers are used for syphilis. Flower stalk are used to treat uterine bleeding and flower receptacles to stop bleeding and to eliminate stagnated blood. Flowers and rhizomes are demulcent, astringent, mild sedative, spasmolytic, antiseptic, used in infusion internally for chronic diarrhea, as a douche for leucorrhea and vaginitis as a gargle for sore throat; also given internally for prostate problems [42]. The flower stalks are used to treat excessive menstruation, bleeding gastric ulcers and postpartum haemorrhage. The lotus honey is used in the treatment of eye infections [43].	Flowers demonstrate hypoglycemic, antipyretic, antidiabetic, antioxidant and free radical scavenging activity, antimicrobial activity, vasodilating effects, antihypertensive and antiarrhythmic abilities. Flowers are rich in aldose reductase inhibitory activity, antiplatelet activity and aphrodisiac activity too[41].	Nornuciferine, N-methylasimilobine, N-methylcoclaurine, isoquercitrin (hirsutrin), dehydroemerine, liriodenine, quercetin, dehydronuciferine, lirinidine, demethylcoclaurine, asimilobine, luteolin glucoside and linalool [44].

*Nerium oleander* Sinhala: Kaneru Tamil: Kanaweeram	This plant is cultivated as a garden plant mostly in North Central province of Sri Lanka.		The ethanolic extract of these flowers has shown a significant antioxidant activity and antimicrobial activity against *Bacillus subtilis*, *Staphylococcus aureus*, *Escherichia coli*, *Salmonella* and *Pseudomonas aeruginosa* and antifungal activity against *Aspergillus niger*, *A. flavus, A. fumigates *and* Rhizopus *species [45]. The dichloromethane extract of the flowers has shown the highest cytotoxic activity against T47D, HepG-2 and K562 cell lines [46].	Alkaloids, flavonoids, tannins, saponins, carbohydrates and phenols.

*Nymphaea stellate* English: Water lily Sinhala: Nil manel		The flowers are used as an astringent, cardiac tonic and febrifuge. Filaments are graded as useful in burning sensations of the body since they possess cooling and astringent properties. These are used to treat bleeding piles and menorrhagia and constitute an ingredient of cooling medicines for cutaneous ailments. Carefully dried fragrant stamens act as diuretic and are used for flavouring tea. Helpful in relieving dryness in the chest. Smelling the flowers is beneficial for persons with warm temperament [5].	It has astringent, diuretic and febrifuge properties. *N. stellate* Wild flowers possess gastro protective action by the suppression of pro-inflammatory cytokines and free radical scavenging activities. It has anti-apoptotic effect against ethanol-induced ulceration. The study suggests that the extract of *N. stellate* flowers has antioxidant, anti-inflammatory, and anti-apoptotic properties [47].	

*Punica granatum* English: Pomegranate Sinhala: Delum		Pomegranate with durva root juice is given to stop bleeding from the nose. The rind of the fruit and flowers combined with aromatics (clove, cinnamon etc.) as astringent in such bowel affection not accompanied with tenesmus. Sour pomegranate: Flower buds bruised are given to relieve fever, cough and bronchitis. The juice is effective in reducing burning sensation in the chest and lessening the blood heat. It is antibilious, stops nausea and vomiting, increases frequency of urine and it's more pronounced than of the sweet variety. Flowers dried and powdered used as tooth powder strengthens the gums and acts as haemostatic for bleeding gums. Fever accompanied by vomiting and diarrhoea is relieved following its use. Also beneficial in jaundice due to heat. The flower of this plant is also used as a remedy for diabetes, diarrhea and vaginal discharge in Unani and Ayurvedic medicine. Also, it is used for the treatment of injuries from falls and grey hair of young men [5].	The alcoholic extract of pomegranate flowers possesses a potent free radical scavenging, antioxidant and hepatoprotective activities. According to this study the alcoholic extract contains a huge amount of polyphenolics and the extract is capable of protecting against oxidative damage to lipids and proteins and also of increasing/maintaining the levels of antioxidant molecules and enzymes *in vivo *[48]. Nasiri *et al. *2015 compared the efficacy of pomegranate flower extract with that of creams containing standard 1% silver sulfadiazine (SSD), base cream and normal saline for treating thermal burn injuries in rats. The results have shown that creams containing the *P. granatum* flower extract have remarkably improved the healing of burn wounds compared with creams SSD [49].	Ethanol extract of pomegranate flowers contains the compounds ellagic acid, 3,3′,4′-tri-O-methylellagic acid, ethyl brevifolincarboxylate, urolic acids, maslinic acids and daucosterol. Also the two flavones, luteolin and tricetin, were found in a methanolic extract of pomegranate flowers [50].

*Rosa damascene* Mill. English: Rose Sinhala: Rosa		Rose buds are more astringent than the full blown flowers and considered cold and dry, cephalic cardical, tonic and aperient, removing biliousness. Buds are considered as astringent, aperient, cardiacal, cephalic tonic, removing bile and cold humours. The rose flower is regarded as refrigerant, tonifying for the vital organs, stomach and intestines, has compound action of causing mild purgation as well as astringency, lessens the heat due to biliousness, gives fragrance is perspiration and reduces its excessive excretion. Externally applied gives relief to warm inflammations and associated pain. The aqua distillate is useful in irritated affections of eyes. Flowers help in curing burning sensations, bud odour from mouth, for improving appetite, relieving headache, toothache (as gargles), stomatitis, beneficially used in abnormal functioning of kidneys, liver, chronic fevers, inflammations and intestinal affections. Rose oil is used as flavoring agents to mask the taste of many obnoxious preparations. A conserve of rose petals with other nutritional ingredients has mild laxative action. It is useful to improve appetite, sore throat, enlarged tonsils and sometimes to relieve common urogenitial disorders and urticarial.	The flowers have cooling, mild laxative, aphrodisiac, antipyretic, cardiotonic and astringent properties.	Volatile oil contains geraniol, citronellol, eugenol. The petals contain vitamin C, quercitrin, quercitannic acid, gallic acid, carotenen and red colouring matter [5].

*Saraca indica* Linn Sinhala: Asoka Tamil: Asogam	Asoka plant grows well in areas like Anuradhapura, Polonnaruwa, Binthannea and Mahiyanganaya etc.	The juice of flowers or syrup is useful cardiotonic as well as brain tonic and relieves dysentery [51]. Flowers are also helpful in treating diabetes, haemorrhoids and for the treatment of uterine disorders in females [52].	The aqueous extract of the flowers has shown antiulcer activity. Also the flower extracts has also shown 50% cytotoxic activity against Dalton's lymphoma ascites and Sarcoma-180 tumour cells without any cytotoxic activity against human normal lymphocytes [53]. The studies have shown that the flower are used to reduce skin tumours induced by 7, 12-dimethyl benzanthracene, to rejuvenate skin complexion, to induce quick healing of skin injuries, and to reduce freckles and external inflammations of the skin since the extracts contain flavonoids [54].	Oleic, linoleic, palmitic and stearic acids, P-sitosterol, quercetin, kaempferol-3-0-P-D-glucoside, quercetin-3-0-P-D-glucoside, apigenin-7-0-p-D-glucoside, pelargonidin-3,5diglucoside, cyanidin-3,5-diglucoside, palmitic, stearic, linolenic, linoleic, leucocyanidin and gallic acid [54].

*Sesbania grandiflora* Sinhala: Katuru murumga	This is a common tree which can be found in the back yard of most of the Sri Lankan homes.		*S. grandiflora* flowers possess anti-cancer, analgesic and antipyretic activities [55]. The phenolic extract of *S. grandiflora *has shown antimicrobial activity against *Staphylococcus aureus*, *Shigella flexneri* 2a, *Salmonella Typhi*, *Escherichia coli* and *Vibrio cholera*. Also, it has shown the growth promoting effect on the common probiotic bacterium *Lactobacillus acidophilus *[56].	

*Sida veronicaefolia* Sinhala: Bevila Tamil: Palampasi	A common plant in the waste ground of low-country in Sri Lanka especially in areas like Batticalo, Haragama etc.	Pale yellow small flowers and unripe fruits are given with sugar in decoction for burning sensation in micturition [27].		

*Sphaeranthus hirtus/indicus* L English: Globe flower/East Indian globe thistle Sinhala: Mudamahana	These plants are grown in the rice field in Sri Lanka. It is common in moist places especially paddy field in the low country like Kurunagala	The leaf, flower and seeds of this plant are ground into a paste and applied topically to treat skin diseases especially in itchings, skin eruptions, eczemas, psoriasis, vitiligo and piles. Also, it is used to treat weak heart, palpitations, joint diseases, liver diseases especially jaundice and in chronic cough. Flowers are useful in promoting eyesight and in the treatment of diabetes. Flowers are highly esteemed as an alternative, refrigerant, tonic, blood purifier and in the treatment of conjunctivitis. Flower paste is given in empty stomach to cure dysentery, diarrhoea and indigestion [57].	*S. indicus* flower extracts have shown the broad spectrum antibacterial activity and they have a significant antifungal and antiprotozoal activity. The flowers also consist of neuroleptic, anxiolytic, hepatoprotective, haemolytic, haemostatic, analgesic and antiarthritic activities. The flower heads are famous for their neuroprotective, antiamnesic, antihyperlipidemic and wound healing activities. Both flowers and the flower heads consist of immunostimulatory activity [58]. Sphaeranthanolide with immunostimulant potential.	Eudesmenolide type of sesquiterpene glycoside, and sphaeranthanolide [57].

*Spilanthes paniculata* Wall. /*Spilanthes acmella* Sinhala: Akmella Tamil: Akkirakaram	The plant is common up to 6000 feet altitude in Sri Lanka	The flower heads of this plant are chewed for toothache [25].	The flower of *S. acmella* is known to produce lipase inhibition properties, diuretic, vasorelaxation, pancreatic properties and antifungal activity especially against *Aspergillus parasiticus, A. niger, Fusarium moniliformi* and *F. oxysporum*. It is found that the extract of flower heads of this plant contains spilanthol which is active against Plutellaxylostella According to studies the aqueous extract of *S. paniculata* flowers has potent diurectic activity. This acts as a loop diuretic, which increases the urinary Na+ and K+ levels [59].	Spilanthol [60].

*Stereospermum suaveolens* (Roxb.)/*Bignonia suaveolens* Roxb Sinhala: Palol Tamill: Ambu/Appu		The flower is used to treat malaria, bronchitis, heart diseases, cancer, purgative, in the treatment of bleeding diseases, diarrhoea of the pitta type and it is good for the throat. The Palol flowers are also used to treat hiccup. These flowers are mixed with honey and given orally [61].		

*Syzygium aromaticum* English: Clove Sinhala: Karabu-neti Tamil: Karuvappu		It allays thirst and nausea in children suffering from worms and ingestion. Along with other ingredients it cures colic, diseases of the chest and throat, cough, hiccough, asthma, diarrhea, urinary diseases and remedy for arthritis and rheumatism [62]. The flower buds of *S. aromaticum* have been used for treatment of male sexual disorders in Ayurvedic and Unani medicines from the ancient times [63].	They consist of carminative, stomachic and stimulant activity. Clove also possesses antiseptic, antibacterial, antifungal, antiviral antiemetic, anti-inflammatory, cytotoxic and anesthetic properties. Clove oil is used for toothache because of its antibiotic and antiseptic properties [63].	Volatile oil, caryophyllin, eugenin, gum, tannic acid and salicylic acid. The main constituent of the volatile oil of clove is eugenol. It also contains minor amounts of eugenol acetate, *β*-cariofilenol, *α*-humulen, *β*-pinene, limonene, farnesol, benzaldehyde, 2-heptanone and ethyl hexanoate [64].

*Tabernaemontana divaricata* Linn English: Crepe jasmine/Pinwheel Flower Sinhala: Wathu sudda	A common garden plant in Sri Lanka.	Crepe jasmine flowers are used to treat corneal inflammation and ophthalmia It is used in Unani medicine for the management of pain. The flowers soaked water is sprinkled on smallpox patients.	In recent investigations it is found out that these flowers possesses Hippocratic screening, antianxiety, anticonvulsant, antidiabetic, cytotoxic, antifertility, anti-inflammatory, antioxidant, and gastro protective effects. The methanolic extract of the *T. divaricate* flowers tested on Wistar rats with chemically induced gastric ulcerations has shown that it possesses gastro protective effects by enhancing the production of the gastric mucosa or preventing its depletion by aggressive factors. Also it was found out that these extracts induce the antioxidant activity in chemically induced gastric ulceration in rats.	Aspidospermatan, corynanthean, ibogan and plumeran sub-types of indole alkaloids [65].

*Vernonia cinerea* Linn English: Ash-coloured Fleabane Sinhala: Monarakudumbiya Tamil: Puvamkurundal	Can be seen in everywhere of Sri Lanka	The flowers are administered for conjunctivitis, fever and rheumatism [24].	According to a study done by Latha *et al* (1998) on arthritis induced rats, it was found out that the flower extract of *V. cinerea* consists of anti-inflammatory property [66].	Alkaloids, flavonoids, saponins, carbohydrates, proteins, phenols, steroids and tannin [66].

*Wrightia antidysenterica* Linn (*Nerium zeylanicum* Linn/*Nerium antidysentericum* Linn) Sinhala: Sudu idda/Wal-idda	Very commonly found in open ground in the low-country, especially near the sea.	Flowers are used for snake bite cures, especially for that of the Russell's Viper [7]. Also these are used to treat gonorrhea, ulcers in genital organs, ailments after delivery [67].		

*Woodfordia floribunda* Sinhala: Militta Tamil: Dhatari-puspam	Grows in open sunny places in the lower mountain region of Sri Lanka.	Flowers are added to “Aristas” to cause alcoholic fermentation. They are also used in dysentery and other bowel complaints, irritant haemorrhage, to treat sprue, rheumatism, dysuria, hematuria and dysmenorrhoea. The powder of the flower is given with honey in leucorrhoea [3]. It is also an ingredient of a preparation used to make barren women fertile. The fresh flowers are used to stop bleeding in emergency cuts, while the dried flower powder is used to heal wounds and oil-based flower extract is used to treat open wounds. Dried flower is also used to treat otorrhoea. Water decoction of the fresh flowers, either alone or in combination with ginger (*Zingiber officinale*) is used for the treatment of dysentery [68].	Flowers have astringent and stimulant activity. When dry they are astringent and tonic. The methanolic extract of the flower was tested on diclofenac induced toxicity in rats and found out that this flower extract possesses hepatoprotective activity and it also reduces the liver fibrosis induced by diclofenac [69]. Also the extract has weak cytotoxic activity and potent anti-inflammatory activity [70]. A study has shown Ayurvedic drug ‘Nimba Aristha', which contains *W. fruticosa* flowers, possesses immunomodulatory activity [71].	Tannic acid, ellagic acid, chrysophanol-8-O-_-dglucopyranoside, pelargonidin 3,5-diglucoside, octacosanol and non-phenolic compounds like sapogenin and hecogenin etc [68].
